# Characterization of changes in the intestinal microbiome following combination therapy with zinc preparation and conventional treatment for children with rotavirus enteritis

**DOI:** 10.3389/fcimb.2023.1153701

**Published:** 2023-09-29

**Authors:** Ning Xu, Wen Zhang, Jingjing Huo, Rui Tao, Taicheng Jin, Yuanmou Zhang, Yanjiao Wang, Lei Zhu, JiaJia Li, Qi Yao, Li Ge

**Affiliations:** ^1^ Department of Clinical Laboratory, The First People’s Hospital of Yunnan Province, The Affiliated Hospital of Kunming University of Science and Technology, Kunming, China; ^2^ Department of Gastroenterology, The First People’s Hospital of Yunnan Province, The Affiliated Hospital of Kunming University of Science and Technology, Kunming, China; ^3^ Department of Reproductive Medicine, The First People's Hospital of Yunnan Province, The Affiliated Hospital of Kunming University of Science and Technology, Kunming, China; ^4^ Department of Government, Hamilton College, Clinton, UT, United States; ^5^ Department of Pharmacy, The First People’s Hospital of Yunnan Province, The Affiliated Hospital of Kunming University of Science and Technology, Kunming, China; ^6^ Department of Emergency Medicine, The First People's Hospital of Yunnan Province, The Affiliated Hospital of Kunming University of Science and Technology, Kunming, China

**Keywords:** rotavirus enteritis, zinc preparation, intestinal microbiome, flora changes, inflammatory factors

## Abstract

**Background:**

Rotavirus (RV) is one of the most common pathogens causing diarrhea in infants and young children worldwide. Routinely, antiviral therapy, intestinal mucosa protection, and fluid supplementation are used in clinic, however this is not efficacious in some severe cases. Zinc supplementation has previously been shown to improve resolution of symptoms from infectious diarrhea.

**Methods:**

In this study differences in response rate, duration of hyperthermia, vomiting, and diarrhea, and the persistence time of cough and lung rales in groups were compared. 16SrDNA gene sequencing technology was used to analyze and compare changes in the intestinal microflora of children with RV enteritis who received the conventional treatment with or without the zinc preparation. In addition, the correlations between the differential bacterial species and the related inflammatory factors were determined.

**Results:**

Conventional therapy combined with the zinc preparation significantly shortened the duration of hyperthermia, vomiting, and diarrhea compared with the conventional treatment alone. In addition, the time to symptom relief showed that the absorption time of cough and lung rales was significantly shorter in the combination treatment group than that in the conventional treatment group in the children with pneumonia. Further, compared with the conventional treatment, the combined treatment significantly increased the diversity and abundances of florae as compared with the conventional treatment. This combination therapy containing zinc preparation markedly increased the abundances of Faecalibacterium, Bacteroidales, Ruminoccoccoccus, and Lachnospiraceae at the genus level. The LEfSe analysis suggested that *Clostridiumbolteae* were most significantly altered after the combination therapy. In addition, a correlation analysis revealed significantly negative correlations between the inflammatory factors especially IL-6, TNF-a, CRP and some intestinal florae such as *Bacteroides, Faecalibacterium, Blautia, Parabacteroides, Subdoligranulum*, and *Flavonifractor.*

**Conclusion:**

Compared with the conventional therapy alone, the combined therapy with the zinc preparation significantly improves symptoms caused by RV. The combination therapy containing the zinc preparation significantly increases the diversity and abundances of some beneficial groups of bacteria. Further, The presence of these groups was further negatively correlated with relevant inflammatory factors. More importantly, this combination therapy containing the zinc preparation provides a reference for the clinical management of children with RV enteritis.

## Introduction

Rotavirus (RV), a double-stranded RNA virus of the family *Eutheroviridae*, was discovered in 1973 (Bishop et al., 1973). It is one of the most common pathogens causing diarrhea in infants and young children worldwide. Most children are infected with RV at least once before 5 years of age (Florez et al., 2020), and it is one of the leading causes of non-bacterial acute gastroenteritis in infants and young children. In addition, RV is one of the leading predisposing factors for acute bacterial gastroenteritis, resulting in an estimated 150,000 deaths per year, mainly in developing countries (Sudarmo et al., 2015; Nahari et al., 2017). Commonly, children with RV enteritis often have varying degrees of related clinical symptoms including high fever, vomiting, diarrhea, and abdominal pain. Although conventional medications such as antiviral therapy, intestinal mucosa protection, and fluid supplementation are widely used in clinic, they can be ineffective for some severe cases (Florez et al., 2020). Thus, it is urgent to explore more effective therapies for RV enteritis.

Routinely, zinc preparation is applied in pediatrics to enhance immune resistance, protect gastrointestinal mucosa, and promote wound healing. Studies have shown that administration of the zinc preparation promotes rapid resolution of symptoms and improves therapy efficacy in children with infectious diarrhea (Wang et al., 2017; Malla et al., 2019).

The gut is a complex ecosystem with multiple biological interactions between the host system and gut microbiota, and this is disrupted during gastroenteritis due to viruses (Hooper et al., 2012; Ashrafian et al., 2021; Li et al., 2022). The gastrointestinal tract is inhabited by a microbial ecosystem of approximately 1,014 species that are crucial in the homeostasis of the host (Kim et al., 2021). The exact link between human RV enteritis and intestinal flora has been investigated previously by analyzing the intestinal microbial populations in the population vaccinated with live RV vaccine (Robertson et al., 2021). A correlation study between serum zinc level and diarrhea suggested that the zinc preparations may regulate the immune system through antioxidant, antibacterial, and anti-inflammatory effects (Alaullah et al., 2010). To date, a small number of studies report the intervention efficacy of the zinc preparation in the RV enteritis patients (Jiang et al., 2016; Cai et al., 2022). However, the specific mechanism of this preparation for RV enteritis remains unclear yet. In order to compare the efficacy of conventional treatment with and without zinc preparation on the intestinal microflora, we selected 45 children with RV enteritis and randomly devided into the three groups (n = 15) including a pretherapy group (Untreated), a conventional treatment group (Treated-1), and conventional treatment combined with zinc group (Treated-2). The intestinal flora was characterized using16S rRNA gene sequencing. Further, the correlations between the differential bacteria and the relevant inflammatory factors were performed. The aim of this study was to characterize the impact of zinc-containing combined treatment on the intestinal microbiome with a view to developing new interventions for treatment of RV enteritis in the clinic.

## Methods

### Therapy for children with RV enteritis

#### Enrollment

A total of 45 children were included in the present study. They were diagnosed as RV enteritis and then hospitalized in the Department of Pediatrics of the First People’s Hospital of Yunnan Province (Kunming, China) between January 2021 to January 2022. Among them, 15 children pretherapy were selected as controls. In addition, 15 received the conventional treatment, and 15 children received the conventional treatment combined with zinc preparation. All the fecal samples were collected 6 h after the fastings. This study was approved by the Ethics Committee of the First People’s Hospital of Yunnan Province, and the relatives of all the children carefully read and signed the informed consent forms.

#### Treatments

The conventional treatment included oral suspension smecta (Bofu Yisheng Pharmaceutical Co., Ltd., Tianjin. 3g/bag). The oral method included 1g tid for children 6 months to 1 year old, 1.5 g tid for children 1 year to 2 years old, and 2 g tid for children 2 years to 3 years old. The conventional treatment lasted for 7 days continuously and once a day.

Beside the conventional treatment described as before, the combination therapy included the additional zinc preparation treatment (zinc gluconate granules, Zhejiang Hangkang Pharmaceutical Co., Ltd., 35mg/bag, equivalent to 5mg zinc). The oral method included 1/3 bag tid for children 6 months to 1 year old, 1/2 bag tid for children 1 to 2 years old, and 1 bag tid for 2 to 3 years old. Also, the combination therapy lasted for 7 days continuously and once a day.

### Inclusion and exclusion criteria

#### Inclusion criteria

(i) diagnosed as acute diarrhea, the duration was less than 48 hours, and ages were 12 months to 3 years;
(ii) positive RV was confirmed by the test of fecal RV antigen after the admission.

#### Exclusion criteria

(i) those with bacteriophage dysentery and/or combined with bacterial enteritis;
(ii) those who received antibiotics or probiotic agents within 2 months prior to enrollment;
(iii) those with recurrent intestinal enteritiss or gastrointestinal symptoms;
(iv) those with heart diseases or immunodeficiency diseases combined with other types of infectious diseases;
(v) those with chronic diseases such as type I diabetes mellitus, leukemia and norovirus infection.

### Sample collection

At least 6 g fresh stools were collected from the subjects in a clean environment and placed in sterile sampling tubes, then sent to the laboratory to store at -80°C for test. The baseline information, past medical history, and dietary habits of the patients were recorded by questionnaires.

### Criteria for judgement of efficacy

The criteria for judgement of efficacy of acute diarrheal diseases were compliant with the National Symposium on Prevention and Treatment of Diarrheal Diseases in 2020 (Expert group for the validation of the preparation of the code of practice for the diagnosis and treatment of acute infectious diarrheal diseases in children (2020). (i) Effective: the numbers, properties of stools, and the systemic symptoms were improved significantly within 72 h after the treatment; (ii) Ineffective: the numbers, properties of stools, and the systemic symptoms were not significantly improved or even deteriorated within 72 h after treatment.

### DNA extraction and 16S rRNA gene amplification and sequencing

DNA was extracted with QIAamp DNA Stool MiniKit according to the manufacturer’s instructions and stored at -20°C for analysis. A series of PCR primers designed by GENEWIZ was used to amplify two highly variable regions of prokaryotic 16SrDNA, including V3 and V4, using 20-30 ng of DNAs as templates. Forward primer “5’-CCTACGGRRBGCASCAGKVRVGAAT-3’” and reverse primer “5’- GGACTACNVGGGTWTCTAATCC-3’” were used to amplify the V3 and V4 regions. The PCR products were purified and subjected to NGS sequencing. Library concentrations were checked with an Infinite 200 Proenzyme labeler (Tecan, Hombrechtikon, Switzerland), and PE250/FE300 double-end sequencing was performed according to the Illumina MiSeq/Novaseq (Illumina, San Diego, CA, USA) instrument instructions. Raw sequencing data (through-filtered data) were obtained. Taxonomic analysis of representative sequences for each operational taxonomic unit (OTU) was performed using the Ribosome Database Project general Bayesian classifier in combination with the 16SrRNA database (SILVA Release138).

### Detection of relevant clinical indices in the blood

C-reactive protein (CRP) and procalcitonin (PCT) were assayed by enzyme-linked immunosorbent assay, interleukin-6 (IL-6) and tumor necrosis factor-α (TNF-α) were measured by scattering turbidimetric assay, endotoxin was detected by photometric assay, and D-lactate was determined by colorimetric assay.

### Bioinformatics analysis and statistics

The NovaSeq PE250 sequencing platform was used to construct a small fragment library based on the characteristics of the amplified V3-V4 region, and the library was double-ended sequenced using the Illumina NovaSeq sequencing platform. The raw data was spliced to sequence, and denoised with DADA2 to obtain each deduplicated amplicon sequence variants (ASVs) (Callahan et al., 2016). The abundance of these ASVs in the samples corresponded to the abundance of OTUs, abandoned low-quality and low complexity sequences to control the quality, and spliced sequence by overlapping relations.

The UPARSE 7.1 software aviable at http://drive5.com/uparse/ was applied to obtain OTUs by clustering rDNA sequence at 97% similarity and removing chimeric sequences (Edgar, 2013). Analysis softwares including Cutadapt (v1.9.1), Vsearch (1.9.6), Qiime (1.9.1) and Cytoscape (3.9.0) were selected in this study. The OTU representative sequences were compared and the species were annotated based on the Greengene database. The confidence threshold was set at 80%.

Based on the OTU analysis, the alpha diversity indices including Chao1, Shannon, and Simpson were calculated to reflect the species abundance and diversity of the community, and rarefaction and rank-abundance curves were plotted to reflect the species richness and evenness.

Principal coordinates analysis (PCoA) and Non-metric multidimensional scaling (NMDS), as well as sample clustering tree, were used to explore the differences in community structure between samples or groups. PCoA was performed based on the sample OTU abundance table. Metastats difference analysis was used to show the OTUs possessing significant differences in abundances between the two microbial communities using rigorous statistical methods by plotting species co-occurrence network. The (un)weighted unifrac analysis was used to compare the differences in the microbial communities between the samples in the two groups.The LEfSE analysis was used to compare the variations in the species between the two groups. In addition, network analysis was used to display the interactions of the microbial communities or species that were varied between the two groups. All the data were analyzed statistically using SPSS17.0 software (Chicago, IL, USA), and *p* < 0.05 is thought to be significant.

## Results

### Baseline information of children with RV enteritis

Forty-five children who met the inclusion criteria were randomly divided into an Untreated group, a Treated-1 group, and a Treated-2 group described as above.

The baseline information of the children with RV enteritis were listed in **Table 1**.

**Table 1:** General information on children included in the study.

Group	n	Age(x̅ ± s, months)	Male/Female
**Untreated**	15	22.9±7.8	8/7
**Conventional**	15	24.0±5.9	7/8
**Combined**	15	23.4±7.0	8/7

### Therapeutic efficacy

It showed that there was a significant difference in the incidence rate of fever among the three groups (χ2 = 6.51, p = 0.04). However, there were no significant differences in the incidence rates of vomiting and light dehydration among the groups (p > 0.05) (**Table 2**).

**Table 2:** Incidence rate (%) of symptoms among the three groups.

Group	Fever [n(%)]	Vomiting [n(%)]	Light Dehydration [n(%)]
**Untreated**	12 (80%)	9 (60%)	8 (53.3%)
**Conventional**	6 (40%)	8 (53.3%)	4 (26.7%)
**Combined**	5 (33.3%)	4 (26.7%)	3 (20%)
**x^2**	6.51	3.13	3.88
**p value**	0.04	0.21	0.14

The results showed that the persistence time of symptoms including fever, vomiting, cough, diarrhea, pulmonary hales was significantly shortened in the Treated-1 and -2 groups compared to the Untreated group (p < 0.01). Compared with the conventional treatment, the combined therapy containing the zinc preparation significantly shortened the persistence time of the symptoms except for vomiting. (**Table 3**).

**Table 3:** Persistence of symptoms in the children with RV enteritis (Day) (x̅ ± s, n =15).

Group	Fever	Vomiting	Cough	Diarrhea	Pulmonary Hales
**Untreated**	4.22 ± 0.94	3.52 ± 0.61	5.17 ± 0.44	6.03 ± 0.61	6.12 ± 0.41
**Conventional**	3.7 ± 0.38	2.64 ± 0.4	4.37 ± 0.41	5.07 ± 0.26	5.17 ± 0.43
**Combined**	2.54 ± 0.67	2.54 ± 0.67	3.13 ± 0.26	4.13 ± 0.37	3.25 ± 0.4
**F value**	22.63	13.33	110.64	71.47	187.44
**p value**	<0.01	<0.01	<0.01	<0.01	<0.01

### OTUs

**Figure 1:**
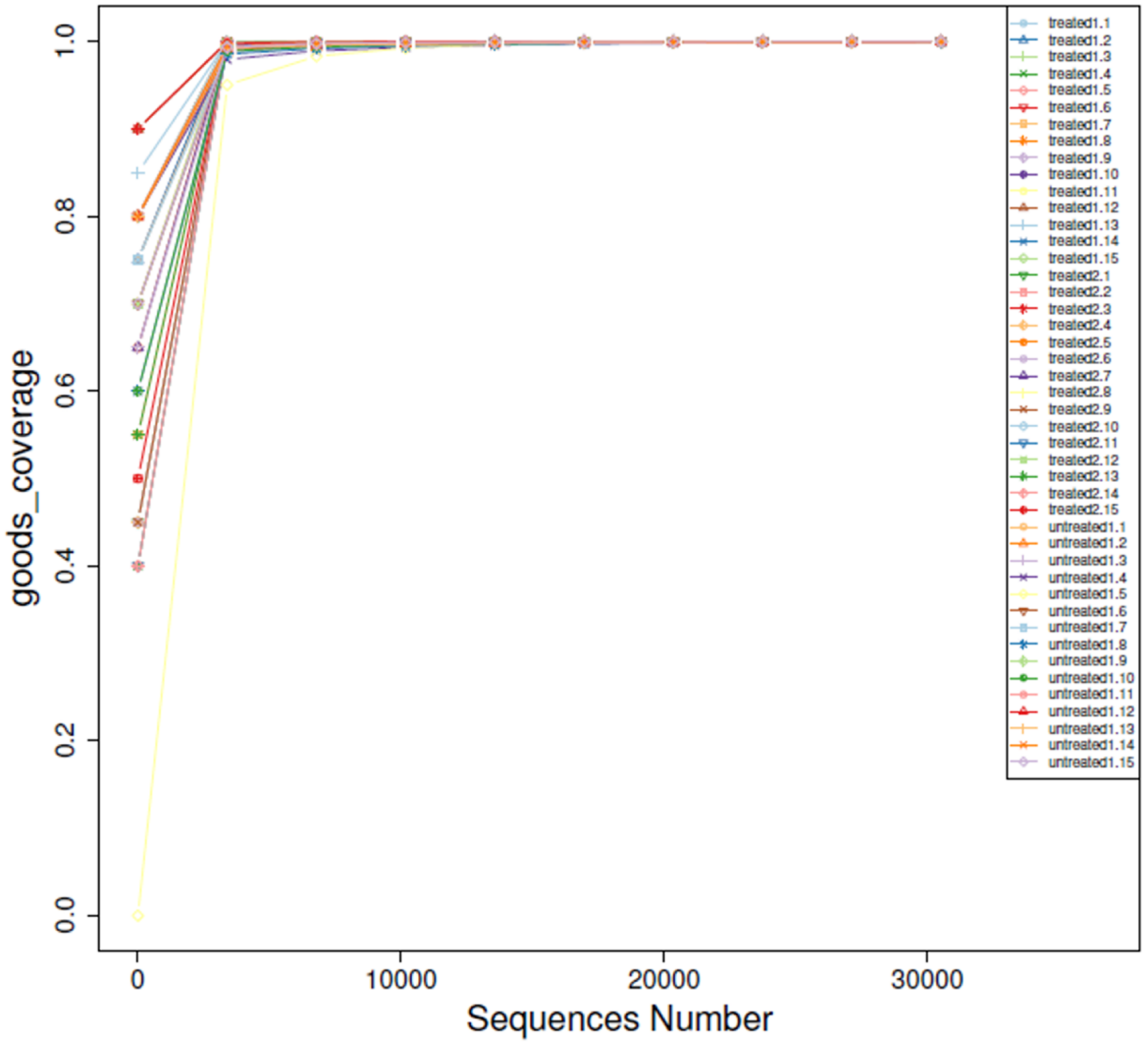
Rarefaction curves of OTUs. The x-axis represents the number of valid sequences per sample, and the y-axis represents the number of observed OTUs.

The sample size increased gradually and eventually stabilized, the number of OTUs detected no longer increased with increase of the extracted data. Rarefaction analysis showed that the dilution curves of the included samples approached saturation, with an index almost equal to 1, indicating that the sequencing was sufficient to cover the vast majority of bacteria, and the sample number was reasonable. In each sample, most of the gut microbial diversity was captured with the current sequencing depth (**Figure 1**).

### α diversity and β diversity of intestinal flora

A total of 45 fecal samples were analyzed using 16sRNA gene sequencing. α and β diversity analysis was performed to assess the difference in the bacterial diversity between the zinc-containing combination treatment group and the conventional treatment group.

**Figure 2:**
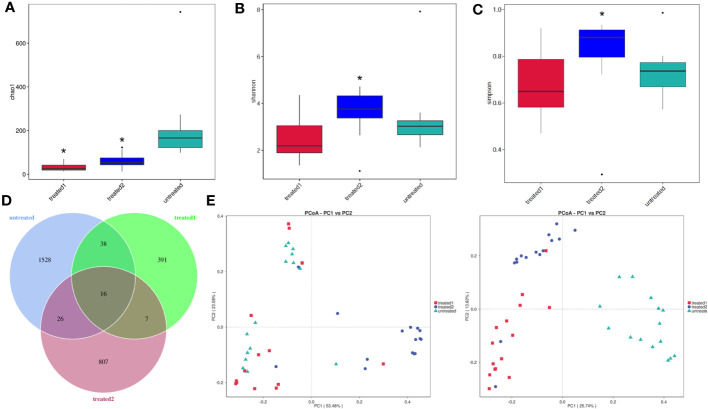
α diversity and β diversity of intestinal flora. **(A-C)** α diversity indices. ^*^ *p*<0.05 vs. untreated. The feces samples were collected and 16S rDNA sequencing was then performed. α diversity indices including Chao1, Shannon, and Simpson were detected to compare the differences among the groups; **(D)** Venn diagram assay. The number of intestinal flora was highest in untreated group, and more than that in Treated-2 group and Treated-1 group. 16 kinds of the intestinal flora were the same in the three groups; **(E)** PCoA analysis. PCoA assay suggested a good sample separation among the three groups.

The results showed that the Chao1 index was higher in the untreated group than that in the Treated-2 and Treated-1 groups (*p*<0.05), which was not in accord with our prediction (**Figure 2A**). The Shannon and Simpson indices were higher in the Treated-2 group than those in the untreated group (*p*<0.05) (**Figures 2B, C**). It suggested that the species diversity in the untreated group was the most abundant, and the zinc-containing combination treatment was the best from evenness of the flora.

A Venn diagram showed that 2813 operational taxonomic units (OTUs) were obtained for all the 45 samples in the three groups.Meanwhile, 1528,391, and 807 OTUs were characteristic the unreated, Treated-1 and Treated-2 groups, respectively. Meanwhile, the three groups shared the same 16 OTUs (**Figure 2D**).

The similarity between the clusters was processed by R language programming using principal coordinates analysis (PCoA) and similarity analysis (Anosim). The Anosim analysis showed R = 0.405 and *p* = 0.005 between the zinc-containing combination treatment and the conventional treatment. The difference between the two groups was greater than that in the intra group, indicating significant differences in the bacterial communities between the two treatment groups. The three groups in this study showed good sample separation after the principal coordinates analysis and similarity analysis, and β-diversity by PCoA showed a clear clustering in the three groups. A significant cohort difference between the three groups could be found, whereas there was a small overlap between the conventional treatment group (Treated-1) and the zinc-containing combination treatment group (Treated-2) (**Figure 2E**).

### Analyses of intestinal flora composition at the phylum, class, order, family, and species levels

**Figure 3:**
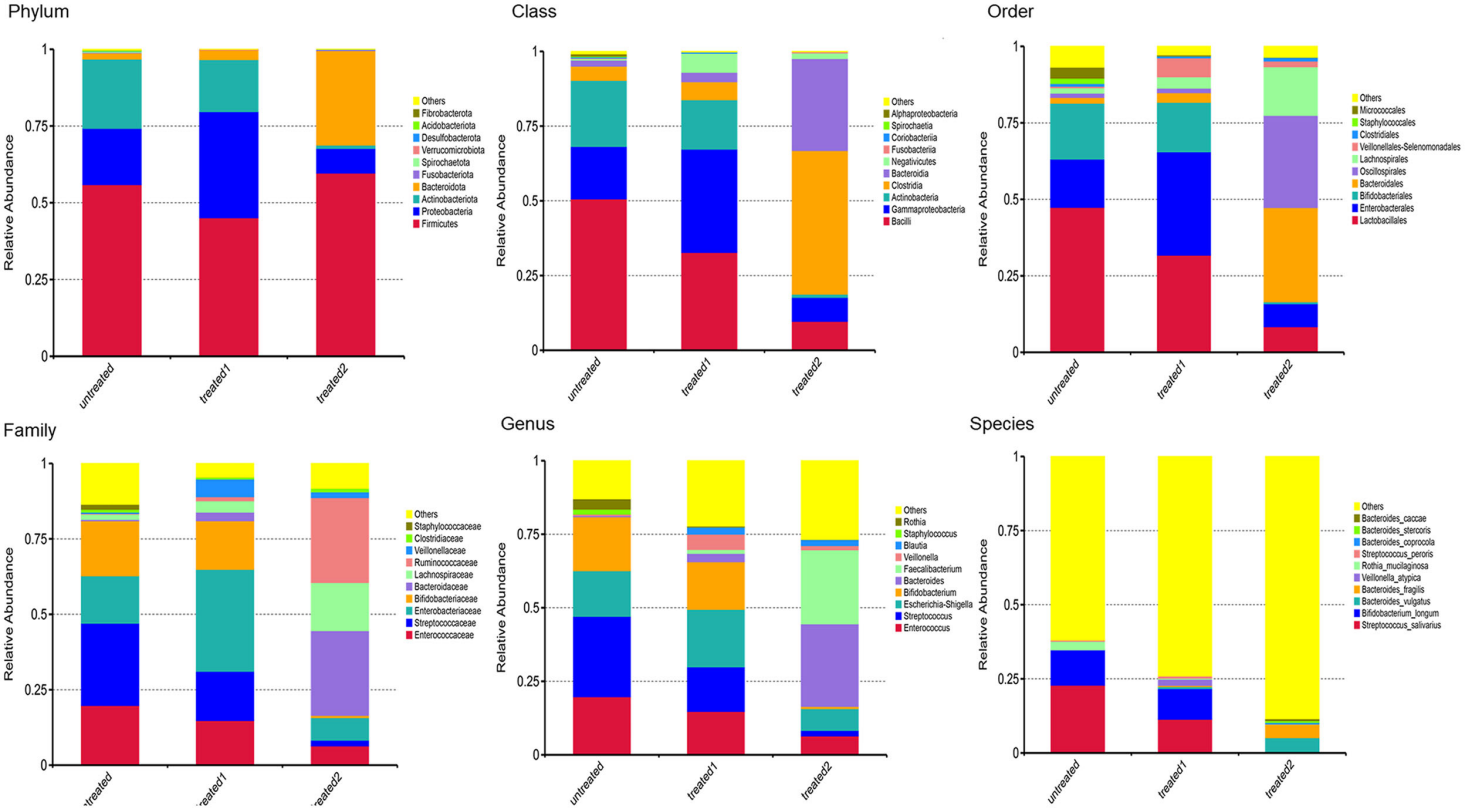
Analyses of intestinal flora composition at phylum class, order, family, and species levels (top 10 bacteria). Changes in flora compositions at phylum, class, order, family, and species levels. The significantly changed flora were shown to reflect their proportions in the community at various levels. The top 10 bacteria were displayed to show the differences of the groups.

The microbial communities at the phylum level were significantly different between the three groups. Compared with the untreated group, *Proteobacteria* was most significantly increased and *Actinbacteria* was slightly decreased in the conventional treatment group, and *Proteobacteria* was significantly increased and *Actinbacteria* was markedly decreased in the zinc-containing combination therapy group (**Figure 3**).

The change trend at the class level was not only continuous but also accelerated compared to the phylum level. The abundance of *Clostridiumbolteae* exceeded *Bacteroidia*, and it became the most significant species after the combination treatment (**Figure 3**).

The species structure diagram at the phylum and class levels showed the conventional treatment continued the bacterial community structure of the untreated group, while the zinc-containing combination conventional treatment sharply increased the abundance of *Bacteroidia*, and added a new bacterial community *Clostridiumbolteae*, resulting in significant reconstruction (**Figure 3**).

At the order level, compared with the untreatment, the conventional treatment slightly increased the abundance of *Bacteroidobacteria*, and significantly increased the abundance of *Enterobacteriales*. Compared with the untreated group, the zinc-containing combination treatment led to a completely different microbial community structure, with a significantly increased abundance in *Bacteroidales*, as well as a significant increase in *Oscillospirales* and *Lachnospirales* (**Figure 3**).

At the family level, compared with the untreatment, the conventional treatment slightly increased the abundance of *Bacteroidaceae* and significantly increased the abundance of *Streptococcaceae*. Compared with the untreatment, the zinc-containing combination treatment significantly increased *Bacteroidales*, as well as a significant increase in a new bacterial family, *Ruminococcaceae*. (**Figure 3**).

At the order and family levels, the species structure diagram showed that the conventional treatment was similar to bacterial community structure of the untreatment. However, the zinc-containing combination therapy greatly increased the abundance of *Bacteroidia*. In addition, new bacterial communities including *Oscillospirales*, *Lachnospirales*, and *Ruminococcaceae* occurred, and the bacterial community structure underwent significant reconstruction (**Figure 3**).

At the genus level, compared with the untreatment, the conventional treatment maintained the microbial community structure of the untreated group, except for a slight increase in the abundance of anaerobic bacteria *Veillonella*. However, the zinc-containing combination treatment produced completely different microbial community structures and significantly increased the abundance of new genus *Faecalibacterium* (**Figure 3**).

Similar to the genus level, at the species level the structure in the conventional treatment group remained basically unchanged compared to the untreated group. The conventional treatment group maintained the microbial community structure in the untreated group except for a slight increase in the abundance of anaerobic bacteria *Veillonella*. However, the zinc-containing combination treatment showed completely different microbial community structures and a significant increase in the abundance of new species *Faecalibacterium* (**Figure 3**).

At the genus and species levels, the combination treatment produced a new bacterial community with differentially more *Faecalibacterium*, reconstructing the bacterial community structure (**Figure 3**).

### Metastats variance analysis

Compared with the untreatment, the conventional treatment significantly increased the abundance of *Veillonella* (*p* = 0.042), while the zinc-containing combination treatment drastically increased the abundance of *Bacteroidia* and *Faecalibacterium* (*p* < 0.001).

**Figure 4:**
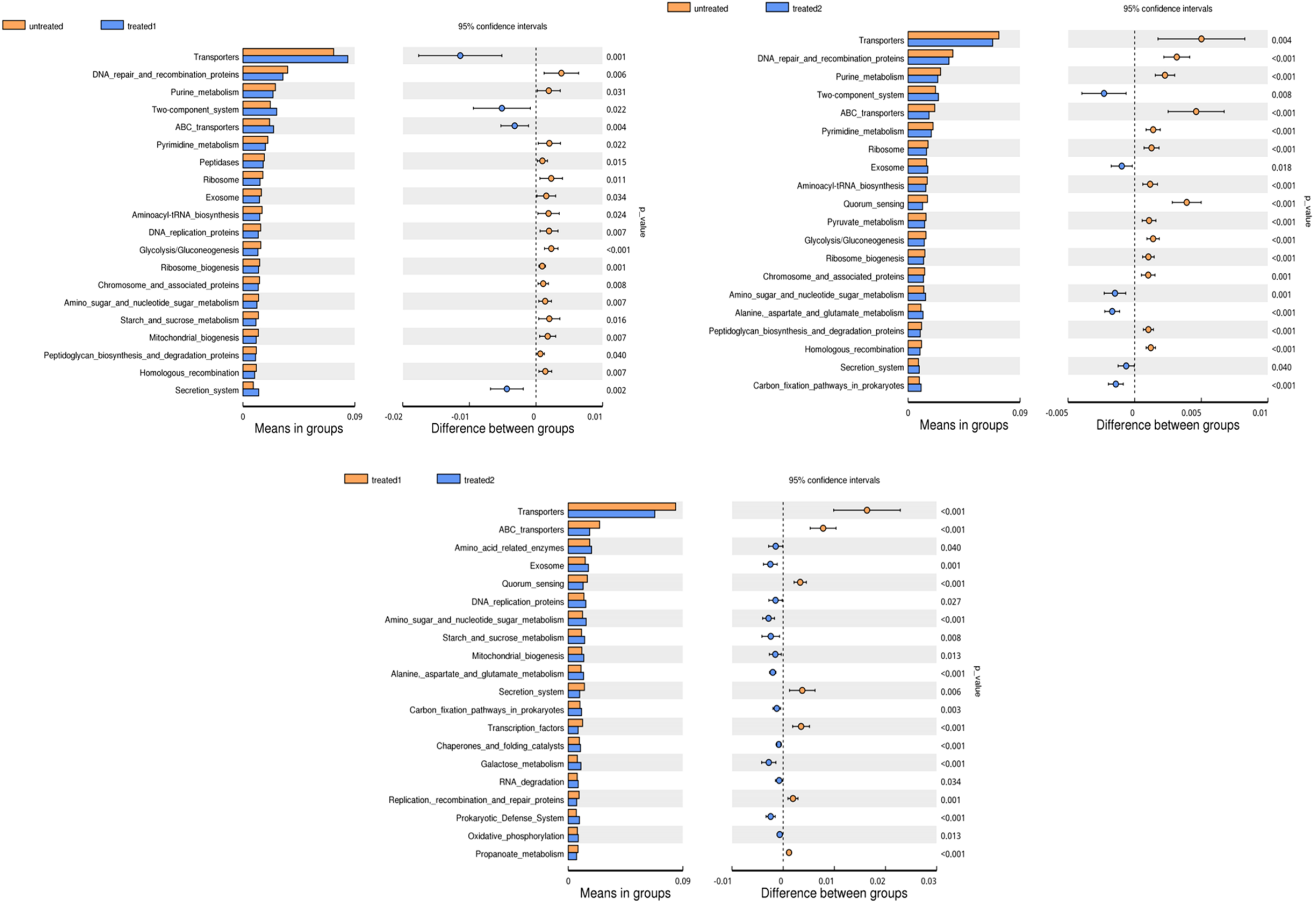
Analysis of species differences between groups. Each bar in the graph represents the mean of species with a significant difference in abundance between the two groups. The right figure shows the confidence level of inter group differences. The left-most point of each circle represents the lowest limit of the 95% confidence interval (95% CI), and the rightmost point of the circle represents the highest limit of the 95% CI. The center of the circle represents the difference in mean, and the color of the circle represents the significance for the inter group differences in the corresponding different species.

Compared with the conventional treatment, the combination treatment significantly reduced the abundance of *Bifidobacterium* (*p* = 0.012) and drastically increased the abundances of *Bacteroidia* (*p*<0.001) and *Faecalibacterium* (*p*<0.001). It suggested that the characteristic florae were *Veillonella* and *Bifidobacterium* in the conventional treatment group, and *Bacteroidia* and *Faecalibacterium* in the zinc-containing combination treatment group (**Figure 4**).

### LEfSe analysis

**Figure 5:**
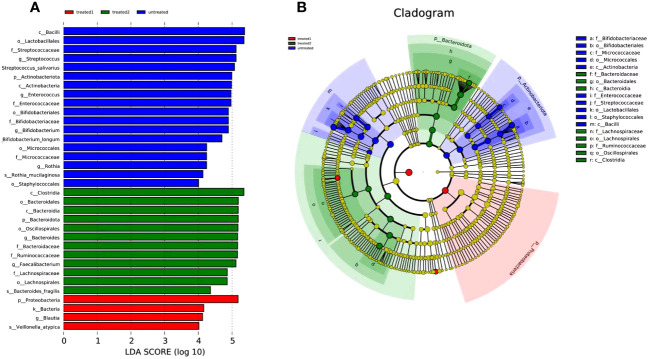
LEfSE analysis of groups. **(A)** tree diagram showing species with significant differences among the three groups; **(B)** evolutionary branch diagram showing the species that are differentially more abundantin the group. Classification is performed from phylum level to genus level. Blue column represents the untreated group, green column represents the zinc-containing treatment group, and red column represents the conventional treatment group.

The LEfSe analysis showed the differences in 34 taxonomic units of the three groups. It displayed classification of species significant influence among the groups. According to the LDA score, *Bacilli* (LDA=5.37) and *Lactobacillales* (LDA=5.35) were the dominant intestinal florae in the untreated group. *Proteobacteria* (LDA=5.18) had the highest abundance in the conventional treatment group, and *Clostridia* (LDA=5.17) and *Bacteroidales* (LDA=5.17) were more abundant in the zinc-containing combination treatment group (**Figure 5**).

### Co-abundance network analysis

**Figure 6:**
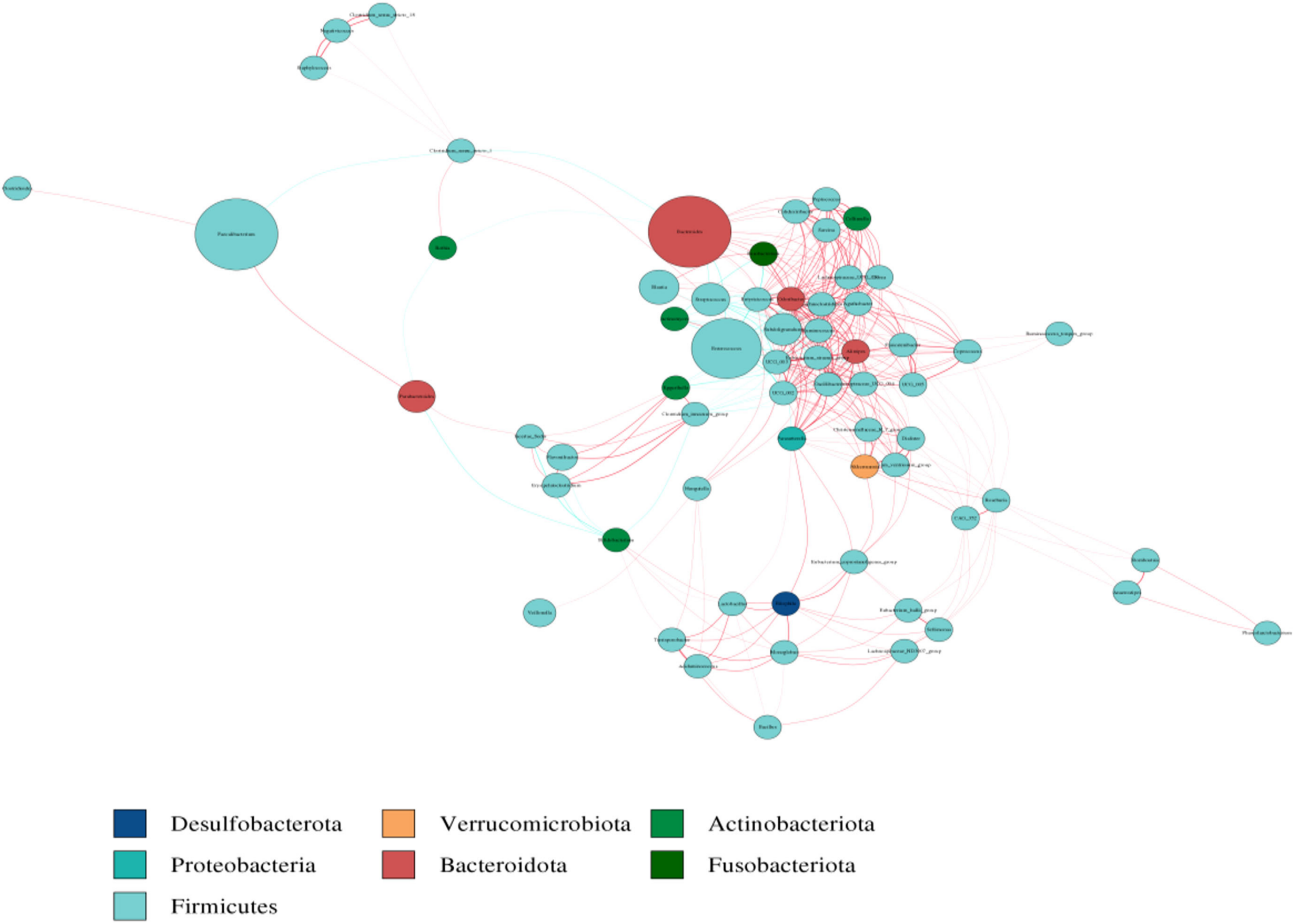
Heatmap analysis of co-abundance network species. Different nodes represent different genera, node size represents the average relative abundance of genus, nodes at same phylum have the same color (shown in the legend), thickness of connecting lines between nodes is positively correlated with absolute value of correlation coefficient between species, and color of connecting lines is positively correlated (red represents a positive correlation, blue represents a negative correlation).

The co-abundance network analysis were conducted using the Cytoscape software to investigate the interactions among dominant species in groups. It showed that *Alistipes*, *Bacteroides*, *Flavonifractor*, *Parasutterell*, *Ruminococcus*, *Lachnospiraceae* had a large number of associations with other microorganisms. Among them, *Alistipes* had the largest associations with other taxa, followed by *Eubacterium* and *Lachnospiraceae*. Some dominant species including *Bacteroides*, *Alistipes*, *Lachnospiraeac*, *Flavonifractor*, *Parasutterell*, *Ruminococcus*, and *Eubacterium* appear to haveunique and important roles in maintaining the structure and function stability of the microbial community after the zinc-containing combination treatment (**Figure 6**).

### KEGG analysis

**Figure 7:**
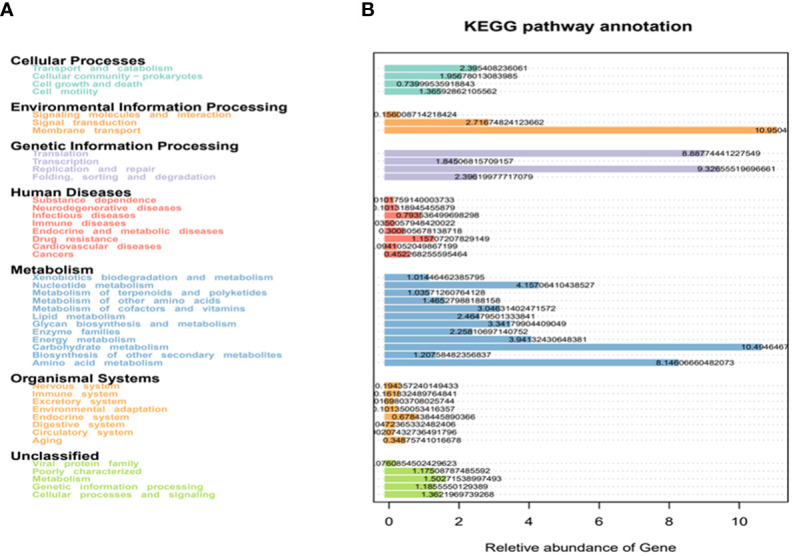
KEGG enrichment analysis. **(A)** the horizontal axis represents the number of differentially expressed genes annotated onto the KEGG pathway, and the vertical axis represents the KEGG pathway; **(B)** heatmap analysis of the functions of the differential expressed genes between the conventional treatment group and the zinc-containing combination treatment group. The red dot represents significantly upregulated gene function, the green dot represents significantly downregulated gene function, the two vertical dashed lines represent log2FC, and the dashed lines represent an adjusted *p* value of 0.05.

The enrichment analysis of the KEGG signaling pathway showed that the dominant species of microbial communities mainly focused on membrane transport and carbohydrate metabolism. Secondly, they were enriched in translation, replication, repair, and metabolic pathways of amino acids in genetic information processing (**Figure 7A**).

In order to evaluate related functions of the differential intestinal florae after the additional zinc intervention, the differentially expressed genes (DEGs) between the conventional treatment group and the combination treatment group were screened by using GO and KEGG enrichment analysis. The heatmap showed that the zinc-containing combination treatment upregulated environmental information processing signaling pathway PI3K-Akt and downregulated environmental information processing membrane_ PTS and human infectious diseases bacteria invading epithelial cells (**Figure 7B**).

### Relationship between community compositions and environmental factors

**Figure 8:**
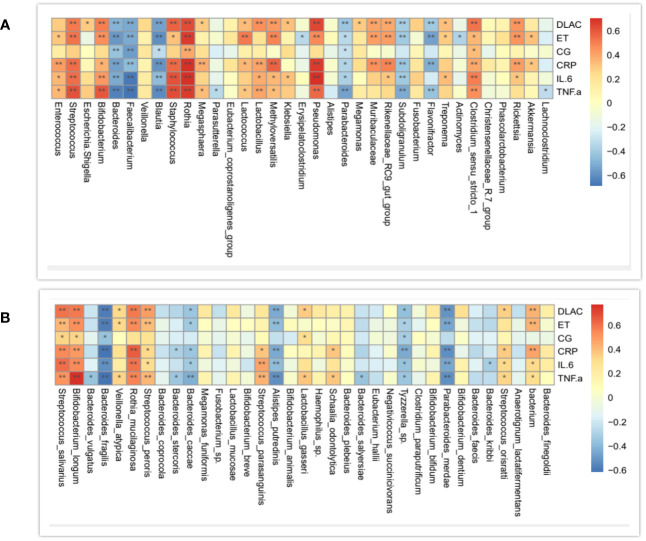
Correlation analysis of community compositions and environmental factors at the genus **(A)** and species **(B)** levels. The correlation is more positive when the correlation analysis is closer to 1, and vice versa. Horizontal axis represents inflammatory factors, and vertical coordinates represents genus names.

In order to investigate whether the zinc intervention change the expression of inflammatory factors in organisms, the correlations between community compositions and inflammatory factors were assayed by the Spearman correlation coefficient analysis.1 At the genus level, the correlation trends of D-lactate, calcitonin, endotoxin, TNF- α, IL-6, and CRP were generally consistent, and TNF- α, IL-6, CRP, and D-lactate had the most consistent trends. Further, *Bacteroides*, *Faecalibacterium*, *Blautia*, *Parabacteroides*, *Subdoliganulum*, and *Flavonifractor* were negatively correlated with the four inflammatory mediators above. In addition, *Clostridium_sensu_stricto_1, Pseudomonas, Rothia, Staphylococcus*, and *Bifidobacterium* were positively correlated with CRP, INF-a, and IL-6 (**Figure 8**).

At the species level, D-lactate, IL-6, TNF-α, CRP and endotoxin had general consistent trends, and the trends of TNF- α, IL-6, CRP, and D-lactate were the closest. *Bacteroides_fragilis, Bacteroides_cacca, Alistipes_putredinis, parabacterium_merdae*, and *Tyzzerella_sp* were negatively correlated with the above four indices. At the species level, there was no specific correlated strain for calcitoningen (**Figure 8**).

In conclusions, compared with the conventional therapy, the combination therapy containing zinc preparation significantly increased the therapeutic efficacy of children with rotavirus enteritis, which might be associated with increased diversity and abundances of some beneficial groups of bacteria, reduced growth and reproduction of harmful bacteria and release of inflammatory factors caused by the zinc preparation.

## Discussion

To the best of our knowledge, this is the first report on the effect of the zinc preparation combined with conventional treatment on the composition of intestinal microorganisms in the children with RV enteritis. That, as we all know, RV enters the body mainly through the gastrointestinal epithelium, and RV is symbiotic with the intestinal epithelial microbiota; therefore, the influence of intestinal microorganisms exists throughout RV enteritis (Zhao et al., 2021).

Clinically, the conventional treatment of RV enteritis includes administration of antidiarrheal drugs such as Natural Montmorillonite powder to relieve diarrheal symptoms, and probiotics such as *Bifidobacterium* and *Bacillus licheniformis* to regulate the intestinal flora and prevent the growth of harmful flora(Azagra-Boronat et al., 2020). A report has shown a better efficacy of zinc preparation combined with probiotics compared with the probiotics alone in the treatment of RV infection. The combination therapy containing zinc significantly increased the overall rate and significantly shortened the time to symptom disappearance as compared with the probiotics treatment alone (Jiang et al., 2016). Also, we found the efficacy of this zinc-containing combination therapy in the present study. This combination therapy was better than the conventional therapy, including higher response rate and shortened persistence of symptoms such as high fever, vomiting, and diarrhea. Also, the study of Jiang has shown that the cardiac enzyme profile indices including CK, CK-MB and AST are significantly reduced after the combination therapy (Jiang et al., 2016). Further, the combination therapy significantly downregulates the levels of inflammatory factors IL-6, IL-8 and hsCRP (Cai et al., 2022). However, until now, it remains unclear how the combination therapy containing-zinc modulate gut microbes in the children with RV enteritis.

In this study, the Chao1 index in the untreated group was the highest, which suggested that the intestinal flora diversity in the untreated patients was the highest. From the bacterial species composition, it showed that the abundances of some pathogenic bacteria including *Enterococcus*, *Streptococcus* and *Escherichia coli* increased sharply, while the anaerobic bacteria in the intestinal tract were almost disappeared and the flora structure was disordered, suggesting that the rotavirus infection caused intestinal flora disorder, destroyed gut mucosal microbial ecosystem, and promoted proliferation of opportunistic pathogens. The conventional treatment corrected the flora disorder and reduced the abundances of harmful and miscellaneous bacteria, but produced no new bacterial strains. The zinc-containing combination treatment effectively inhibited the proliferation of conditional pathogenic bacteria to maintain the stability of the gut microbiota ecosystem, and significantly increased the abundances of *Bacteroidia*, *Clostridiumbolteae*, *Oscillospirales*, *Lachnospirales*, *Ruminococcaceae*, and *Faecalibacterium* which were the characteristic and crucial anaerobic bacteria. Except for *Oscillospirales*, the metabolites of other bacteria above were all short chain fatty acids (SCFAs).

Recently, a correlation analysis showed that they were core commensal bacteria in the intestine and their abundances were negatively correlated with Crohn’s disease, ulcerative colitis and irritable bowel syndrome. Also, it showed that the high abundance of *Faecalibacterium* was sufficient to enable low-titer IgA to produce anti-RV or -NoV effects (Rodríguez-Díaz et al., 2017). In addition, human milk oligosaccharides shortened the duration of diarrhea in RV-infected piglets by increasing the abundance of *Lachnospiraceae* (Li et al., 2014). It has been demonstrated that short-chain fatty acids (SCFAs) produced by intestinal colonies not only maintain the integrity of the intestinal barrier system, but also inhibit intestinal inflammation by modulating the leukocyte recruitment (Parada et al., 2019). For example, SCFAs reduce the products of pro-inflammatory cytokines and mediators INF-a, IL-6 and NO and suppress inflammatory cytokines IL-2, IL-6 and TNF-a secreted by inflammatory cells (Milo et al., 2002).

Next, a correlation analysis is performed between the community composition of intestinal florae and the relevant inflammatory factors. The results revealed that the changes in the trends of TNF-α, IL-6, and CRP were basically consistent in the zinc-containing combination treatment group. It demonstrated that *f_Ruminococcaceae*_Unclassified, [*Ruminococcus*]_gnavus_group, *Flavonifractor Faecalibacterium*, *Lachnospiraceae* were negatively correlated with the intestinal flora. It suggested that these species may play important in relieving diarrhea and reducing inflammation in the children. Notably, SCFAs were associated with themetabolites of these species (Liu et al., 2019).

Among them, the main metabolites of *Bacteroidia* are butyric acid and propionic acid. Currently, a study has confirmed that *Bacteroidia* inhibits the activation of the NF-kB pathway and plays an anti-inflammatory role in IL-10-producing T cells (Ji et al, 2021).

The metastats difference analysis suggested that the main metabolite of *Faecalibacterium* was the butyrate of SCFAs that activated the G protein-coupled receptor CPR41/CPR43 and epigenetic effects to inhibit histone deacetylase, which promoted intestinal barrier protection (Kim et al., 2013). Valeric acid, a main metabolite of *Flavonifractor*, was significantly altered after the combination treatment, and it effectively reduced intestinal mucosal inflammation. An experimental study has demonstrated that a reduction in *Flavonifractor* is a sensitive marker for intestinal inflammation (Gao et al., 2021). A recent study has revealed that *Lachnospiraceae* is effective against host organism infections caused by *Salmonella*, and butyric acid is also the main metabolite of *Lachnospiraceae* (Borton et al., 2017).

The LEfSe analysis found that the abundances of C_ *Clostridiumbolteae* and *Bacteroides* has significantly increased, and these two strains are important in combination therapy. Previously, a study showed that *Bacteroides* and C_ *Clostridiumbolteae* can protect infants from *Salmonella* and *Escherichia coli* infection. They play a protective role in infants. Propionate in their metabolites can prevent Salmonella from starting abnormal cellular functions by reducing the pH value in cells and prolonging the time of first cell division (Brück et al., 2003). Similarly, our research results indicated that C_*Clostridiumbolteae* and *Bacteroides*, two bacteria producing SCFAs, played an immunomodulatory role in RV enteritis, which is consistent with previous reports (Mengel et al., 2012; Kim et al., 2015). Meanwhile, network analysis shows that *Alistipes* under the *Bacteroides* phylum have the greatest connections with other microorganisms, indicating the core role of *Bacteroides* in the zinc-containing combination therapy.

A limtation of this study was the lack of a normal healthy controls group. In future studies, it would be interesting to assess whether additional zinc supplementation shifts diversity towards the normal range, or whether the zinc supplementation induces significant differences in diversity, richness, and composition of the intestinal flora between groups.

In the present study, the additional zinc preparation intervention reshaped the structure of the intestinal flora and produced a large number of SCFAs-producing flora, which might regulate the host immune response to RV through the metabolic pathway of the fatty acids.

The KEGG enrichment analysis showed that the related functions of the differential intestinal flora mainly focused on membrane transport and carbohydrate metabolism pathways after the additional zinc intervention. In environmental information processing, the differential genes were mainly associated with downregulated phosphate transaminase membrane transportation system (PTS) which was responsible for the specific active transport of glucose from the extracellular membrane into cells, and phosphorylating glucose to glucose-6-phosphate in the glycolytic pathway. It suggested that the zinc preparation-induced SCFAs that in turn inhibited the carbohydrate metabolism pathway of harmful bacteria by downregulating the function of PTS in membrane transport.

At the same time, the downregulated genes including the functions of infectious bacteria invading epithelial cells, which may have inhibited the growth and reproduction of harmful bacteria. Further, the differential genes upregulated of the PI3K-Ak signal transduction pathway in environmental information processing. Currently, a study has found that multiple host and probiotic metabolic networks participate in human rotavirus (HRV) diarrhea in neonatal gnotobiotic pigs by using metabolomics analysis (Nealon et al., 2017).Recently, studies have shown that the increased activation of the PI3K/Akt/mTORC1 pathway inhibits macrophage autophagy, reduces plaque macrophage infiltration, and inhibits NF- κB and inflammatory reaction to stabilize vulnerable atherosclerotic plaque (Tian et al., 2014; Zhou et al., 2019), which is consistent with the negative correlations between the increased SCFAs- produced bacteria and inflammatory factors after the zinc intervention in the present study. It provided some evidences for the zinc preparation intervention reshaping the structure of the destroyed intestinal flora after the RV infections. Also, it pointed out some related metabolism pathways to regulate the immune reactions after the RV infections. In addition, it suggested that the SCFAs pathway may be a new and effective therapeutic target for treating rotavirus enteritis. However, the specific pathway needs confirmation by targeting the metabolome in the future study.

## Data availability statement

The data presented in the study are deposited in the figshare repository, accession number is 10.6084/m9.figshare.24147222.

## Ethics statement

The studies involving humans were approved by Ethics committee of First People’s Hospital of Yunnan Province. The studies were conducted in accordance with the local legislation and institutional requirements. Written informed consent for participation in this study was provided by the participants’ legal guardians/next of kin. Written informed consent was obtained from the individual(s), and minor(s)’ legal guardian/next of kin. No potential identifiable images of the participants were included in this article.

## Author contributions

NX designed this study. WZ and LG collected the data. All the authors analyzed and interpreted the data. NX and QY prepared the original manuscript. All the authors participated critical revision of the manuscript. RT, MZ, JW, LZ, and JL collected the fecal samples. TJ performed the statistical analysis. All the authors read and agree the final version of the manuscript.
